# Do modular tapered long stems differ in stability and function? A prospective comparison of two stems in complex femoral revision THA

**DOI:** 10.1186/s10195-026-00925-7

**Published:** 2026-05-04

**Authors:** Mahmoud Fahmy, Mostafa Ahmed Shawky

**Affiliations:** Orthopaedic Surgery, Pelvis Fracture and Arthroplasty Unit, Orthopaedic Department, Kasr Alainy Hospital, Cairo University, Cairo, Egypt

**Keywords:** Revision total hip arthroplasty, Femoral revision, Modular tapered fluted stem, Stem subsidence, Osseointegration, Implant survivorship

## Abstract

**Background:**

Revision total hip arthroplasty (rTHA) in the setting of substantial proximal femoral bone loss is technically challenging. Modular tapered fluted stems provide predictable diaphyseal fixation while allowing independent adjustment of version, offset, and limb length. Among these, two commonly used systems—modular tapered fluted stem Type A (Revitan^™^) and Type B (LIMA Modulus^™^)—have limited direct comparative evidence. This study aimed to prospectively compare radiographic stem subsidence (primary outcome), as well as functional outcomes, complications, survivorship, and secondary outcomes, between Type A and Type B modular long stems in femoral rTHA.

**Methods:**

In this single-center randomized prospective study, 110 patients undergoing femoral revision rTHA were randomly assigned to receive either Type A (*n* = 55) or Type B (*n* = 55) stems. All procedures were performed by experienced revision surgeons under standardized perioperative and rehabilitation protocols. Radiographs were analyzed for stem subsidence, osseointegration, and limb-length restoration. Functional outcomes were assessed using the Harris Hip Score (HHS), Oxford Hip Score (OHS), and European Quality of Life Visual Analogue Scale (EQ-VAS) at baseline and final follow-up (mean 61.4 months). Complications and stem survivorship were recorded prospectively. Statistical analysis included paired and unpaired comparisons, correlation, regression, and Kaplan–Meier survival estimates.

**Results:**

Baseline demographics and femoral defect severity were comparable. Both groups achieved high radiological stability, with mean distal subsidence of 1.3 ± 0.7 mm (Type A) and 1.5 ± 0.9 mm (Type B; *p* = 0.24), and osseointegration in > 92% of cases. Limb-length and offset restoration were similar. HHS improved significantly in both groups (Type A: 44.7 → 88.1; Type B: 45.1 → 87.3; *p* < 0.001), with > 80% achieving good-to-excellent outcomes. Complication rates were low and comparable. Five-year stem survivorship was 98.2% (Type A) and 97.6% (Type B). Early full weight-bearing and lower Paprosky defect grades independently predicted superior functional outcomes, whereas stem type did not.

**Conclusions:**

Both Type A and Type B modular tapered fluted stems demonstrated durable fixation, minimal subsidence, low complication rates, and excellent mid-term functional recovery. Radiographic stem subsidence did not differ between groups, indicating that design variations do not significantly affect clinical outcomes. These findings support the use of modular tapered fluted stems as reliable solutions in complex femoral rTHA.

## Introduction

Revision total hip arthroplasty (rTHA) remains a demanding procedure, especially in the presence of significant proximal femoral bone loss and deficient metaphyseal support [1–4]. The need for revision surgery most commonly arises from aseptic loosening, periprosthetic joint infection, periprosthetic fracture, instability, and polyethylene wear, which collectively account for the majority of rTHA procedures reported in large joint registry analyses and epidemiological studies [5–10]. In these scenarios, modular tapered fluted stems have become a cornerstone of contemporary reconstructive strategies due to their ability to achieve predictable diaphyseal fixation while allowing independent adjustment of version, offset, and limb length [10–15].

The concept of modularity refers to the ability to assemble the femoral component from separate interchangeable segments, typically consisting of a distal fixation stem and a proximal body or neck segment [15–20]. This modular architecture allows surgeons to independently adjust femoral anteversion, limb length, and offset intraoperatively, thereby restoring hip biomechanics despite distorted anatomy or bone loss frequently encountered in revision surgery. Their modularity provides technical flexibility, although differences in taper, modular junction, and surface characteristics may influence early subsidence, proximal bone remodeling, and long-term fixation [20–25].

Among the widely used options, two modular tapered fluted stem platforms—modular tapered fluted stem Type A (Revitan^™^, Zimmer Biomet, Warsaw, IN, USA) and Type B (LIMA Modulus^™^, Lima Corporate, San Daniele del Friuli, Italy)—share the same guiding principle of diaphyseal fixation but differ in taper configuration, proximal body options, and surface technology [19–30]. In general terms, Type A stems employ a fluted titanium stem design available in straight or curved configurations with longitudinal ribs and modular distal–proximal junctions, intended to allow extensive correction of femoral version and limb length in complex revisions. In contrast, Type B stems incorporate a tapered conical geometry with longitudinal flutes and a modular proximal body connected through a Morse-type taper junction, designed to enhance rotational stability and facilitate gradual load transfer along the diaphysis while allowing adjustment of offset and version. Additionally, Type A systems offer curved distal stem options to accommodate femoral bowing, whereas Type B stems rely primarily on conical press-fit fixation with progressive diaphyseal engagement. These design variations may theoretically influence mechanical stability, stress distribution, and early subsidence behavior. Although each design has been individually validated in multiple revision settings, the literature remains largely limited to single-system case series. Direct head-to-head comparisons are scarce, leaving surgeons without robust evidence on whether specific modular architectures translate into measurable differences in subsidence control, diaphyseal integration, complication patterns, or overall survivorship [20–30].

This lack of comparative data represents a clear gap in the current evidence base, particularly given the rising volume of complex femoral revisions worldwide. Understanding whether these systems perform equivalently—or whether design variations confer meaningful clinical advantages—would assist surgeons in selecting the most appropriate implant for complex revision scenarios.

Therefore, this study aims to provide a direct comparative evaluation of Type A and Type B modular tapered fluted stems, assessing radiological stability, subsidence behavior, complication profile, and functional recovery under standardized perioperative conditions. The primary endpoint of the study was radiographic stem subsidence as an indicator of implant stability, while secondary endpoints included functional outcomes (Harris Hip Score), complication rates, and implant survivorship. Rather than formally testing equivalence, the objective of this study is to compare clinical and radiological outcomes between the two systems to determine whether meaningful differences exist in mid-term performance. We hypothesize that both stems will demonstrate comparable mid-term stability and survivorship due to their shared principle of diaphyseal fixation, with subtle design differences potentially affecting early subsidence and proximal bone remodeling.

## Patients and methods

This prospective comparative study was conducted at a high-volume tertiary center specializing in complex hip reconstruction and revision arthroplasty. Ethical approval was obtained prior to commencement, and the study adhered to the principles of the Declaration of Helsinki. All participants provided written informed consent for surgical intervention, data collection, and follow-up evaluation.

Patients undergoing femoral revision total hip arthroplasty with modular cementless long stems were prospectively enrolled between January 2012 and January 2017. All surgeries were performed by senior surgeons with substantial experience in revision arthroplasty. Eligibility criteria included revision of the femoral component for aseptic loosening, mechanical failure, periprosthetic fracture, or second-stage reimplantation following resolved periprosthetic joint infection, with a minimum follow-up of 4 years and complete radiological and clinical records. Exclusion criteria included cemented femoral revision, isolated acetabular procedures, oncologic reconstruction, and insufficient follow-up.

Patients were randomly assigned to receive either modular tapered fluted stem Type A (historically Revitan^™^) or Type B (historically LIMA Modulus^™^) using a computer-generated randomization sequence with block sizes of ten, stratified by Paprosky defect type. Allocation concealment was ensured by sealed opaque envelopes opened immediately before implantation.

Baseline demographic and clinical data—including age, sex, body mass index, comorbidities, and indication for revision—were systematically documented. Laboratory assessment included routine hematologic and biochemical tests, C-reactive protein, and erythrocyte sedimentation rate. In patients previously treated for infection, eradication was verified through joint aspiration with negative cultures. Standardized anteroposterior (AP) pelvic and lateral femoral radiographs were obtained preoperatively, and femoral bone defects were categorized according to the Paprosky classification based on imaging and intraoperative confirmation. Functional status was assessed using the Harris Hip Score (HHS), Oxford Hip Score (OHS), and European Quality of Life Visual Analogue Scale (EQ-VAS).

All procedures were performed under spinal or general anesthesia with patients positioned laterally. A posterolateral approach was used in all cases. An extended trochanteric osteotomy (ETO) was selectively performed to facilitate stem extraction or address substantial metaphyseal deficiency, particularly in Paprosky IIIA–IIIB defects. Following implant removal and thorough debridement, the femoral canal was prepared with sequential reamers and broaches.

**Fig. 1:**
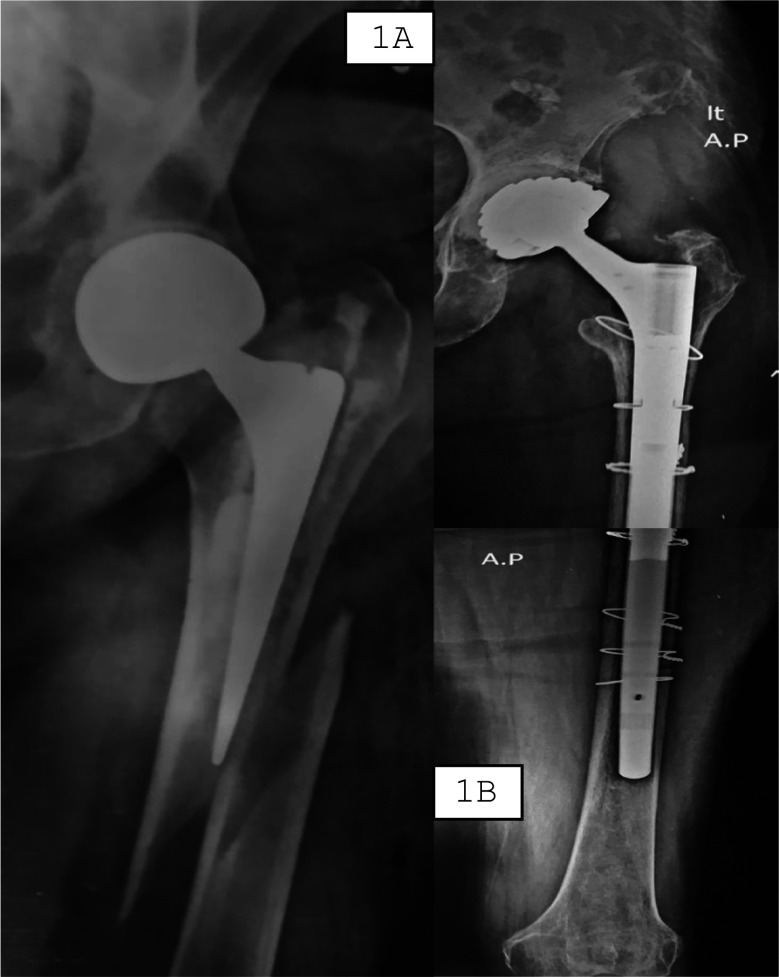
A 58-year-old male patient who underwent revision total hip arthroplasty using a modular cementless long stem (Revitan^™^) for a periprosthetic femoral fracture. **A** AP radiograph of the left hip and femur showing the fracture pattern prior to revision. **B** Final follow-up AP radiograph demonstrating stable fixation, satisfactory alignment, and successful integration of the modular long stem

**Fig. 2:**
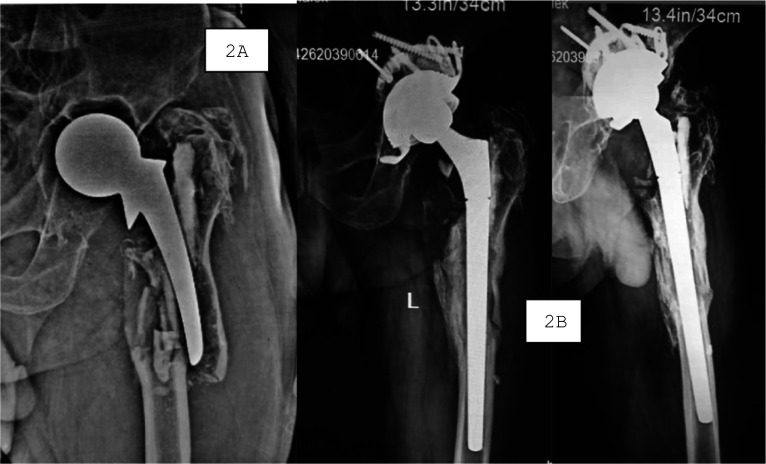
A 62-year-old male patient who underwent revision total hip arthroplasty using a modular cementless long stem (LIMA^™^) for a periprosthetic femoral fracture. **A** AP radiograph of the left hip and femur showing the fracture pattern prior to revision. **B** Final follow-up AP radiograph demonstrating stable fixation, satisfactory alignment, and successful integration of the modular long stem

Both modular systems—Type A and Type B—were inserted using a diaphyseal fixation strategy, targeting a minimum of 5 cm distal fixation for Paprosky type II defects and at least 7 cm for type III defects. Modular junctions allowed adjustment of anteversion, offset, and leg length. Distal locking screws were not used. Fluoroscopic confirmation of trial reduction ensured appropriate restoration of biomechanics before final impaction. Cerclage wiring was applied when necessary to stabilize osteotomies or cortical fissures. Acetabular revision was performed when indicated, using cemented or uncemented components according to bone quality and stability (Figs. 1 and 2 are case examples).

Postoperative management included layered closure and routine drain removal within 48 h. A standardized rehabilitation protocol initiated early strengthening exercises on postoperative day 1. Partial weight bearing began on day 3 and progressed to full weight bearing once radiographic signs of stability or ETO consolidation were observed, typically between 6 and 8 weeks. Chemical thromboprophylaxis was administered for 4 weeks, and antibiotic prophylaxis continued for the first 48 h.

Serial radiographs were obtained at 6 weeks, 3 months, 6 months, 1 year, and annually thereafter. Subsidence was measured as the change in vertical distance between the greater trochanter and a reproducible point on the stem’s proximal body. Subsidence > 2 mm was considered significant, on the basis of prior literature and clinical relevance [2, 3, 5, 6]. Osseointegration was evaluated on the basis of bridging trabeculae, cortical hypertrophy, and absence of progressive radiolucency across Gruen zones. Limb length and femoral offset were compared with the contralateral side when applicable. ETO union was confirmed by cortical continuity and disappearance of osteotomy lines.

All radiographs were independently reviewed by two experienced orthopedic surgeons blinded to stem type and functional outcomes. Radiographs were measured digitally using PACS software with calibration to minimize magnification errors. Inter- and intraobserver reliability were assessed using intraclass correlation coefficients, with values above 0.80 indicating excellent agreement. Missing radiographic or functional data, which represented approximately 5% of total data, were handled using multiple imputations. Sensitivity analysis using complete cases confirmed consistent results.

Functional scores (HHS, OHS, EQ-VAS) were recorded preoperatively, at 6 months, at 1 year, and at final review. The primary endpoint of the study was radiographic subsidence. Secondary endpoints included functional outcomes (HHS, OHS, EQ-VAS), complications, and stem survivorship. Minimal clinically important differences (MCID) were considered for HHS (8 points), OHS (5 points), and EQ-VAS (10 points) to interpret functional outcomes [2, 3, 6]. Complications including dislocation, infection, significant subsidence (> 2 mm), and re-revision were documented prospectively. Subgroup analyses examined revision indication, osteotomy status, and stem type. Additional correlations were explored between subsidence, time to full weight bearing, Paprosky defect severity, and final functional outcomes.

**Fig. 3:**
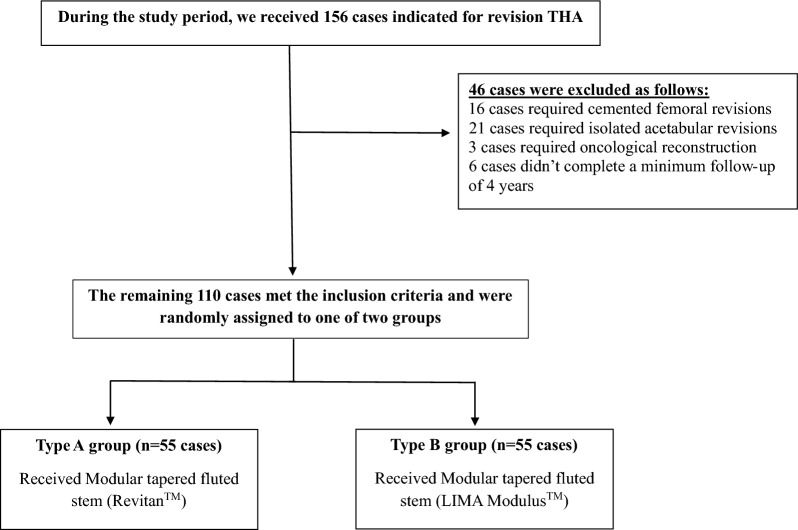
Study flow diagram illustrates the number of femoral revision procedures performed during the study period, including patient exclusions, group allocation, and completion of follow-up

A flow diagram (Fig. 3) illustrates the total number of femoral revisions performed during the study period, exclusions, randomization, and follow-up completion.

Statistical analysis was performed using SPSS v26. Data normality was evaluated with the Shapiro–Wilk test. Continuous data are presented as mean ± standard deviation (SD), and categorical data as *n* (%). Between-group comparisons used *t*-tests or Mann–Whitney *U* tests and chi-squared/Fisher’s exact tests. Pre- and postoperative changes were assessed with paired *t*-tests, correlations with Pearson or Spearman coefficients. Logistic and multivariable linear regression identified predictors of poor outcome (HHS < 80) and determinants of final HHS, adjusting for confounders. Stem survivorship was estimated using Kaplan–Meier analysis. Significance was set at *p* < 0.05, and effect sizes (Cohen’s *d*) with 95% confidence intervals (CIs) are reported. A formal a priori sample size calculation was not performed and acknowledged as a limitation of the study, as all eligible patients during the study period were consecutively included. The resulting cohort of 110 patients is comparable to those reported in previous studies of modular femoral revision stems [2, 3, 6]. On the basis of observed variability in stem subsidence and functional outcomes, post hoc assessment suggests that the sample size was sufficient to detect clinically meaningful differences in primary radiographic outcomes, although the study may remain underpowered for detecting very small differences or rare complications.

## Results

A total of 110 femoral revisions were analyzed: 55 received Type A and 55 Type B modular tapered fluted stems. Baseline demographics were comparable. The Type A group included 30 men (54.5%) and 25 women (45.5%), mean age 67.9 ± 8.8 years; the Type B group included 31 men (56.4%) and 24 women (43.6%), mean age 68.5 ± 9.1 years. Body mass index, comorbidities, follow-up duration (mean 61.4 months, range 48–96), and Paprosky defect distribution were similar between groups. Extended trochanteric osteotomy (ETO) was performed in 15 Type A (27.3%) and 16 Type B (29.1%) cases (Table 1).

**Table 1:** Demographic and preoperative characteristics

Variable	Group A (*n* = 55)	Group B (*n* = 55)	Cohen’s *d*	95% CI	*p*-Value
Age (years), mean ± SD	67.9 ± 8.8	68.5 ± 9.1	−0.07	−0.44 to 0.31	0.68
Male sex, *n* (%)	30 (54.5)	31 (56.4)	—	—	0.83
Female sex, *n* (%)	25 (45.5)	24 (43.6)	—	—	0.83
BMI (kg/m^2^), mean ± SD	28.5 ± 3.7	28.1 ± 3.9	+0.11	−0.27 to 0.48	0.52
CCI, mean ± SD	3.1 ± 1.2	3.0 ± 1.1	+0.09	−0.29 to 0.46	0.71
Follow-up (months), mean ± SD	61.2 ± 10.5	61.6 ± 11.1	−0.04	−0.41 to 0.34	0.81
Revision indication, *n* (%)					
- Aseptic loosening	26 (47.3)	27 (49.1)	—	—	0.84
- Periprosthetic fracture	17 (30.9)	15 (27.3)	—	—	0.65
- Post-infective reimplantation	8 (14.5)	9 (16.4)	—	—	0.79
- Mechanical failure/subsidence	4 (7.3)	4 (7.3)	—	—	1.00
- Vancouver B2 fracture, *n* (%)	13 (76.5% of fractures)	10 (66.7% of fractures)	—	—	0.55
Paprosky femoral defect, *n* (%)					
- Type II	28 (50.9)	29 (52.7)	—	—	0.84
- Type IIIA	15 (27.3)	14 (25.5)	—	—	0.82
- Type IIIB	12 (21.8)	12 (21.8)	—	—	1.00
Extended trochanteric osteotomy, *n* (%)	15 (27.3)	16 (29.1)	—	—	0.83

BMI, body mass index; CCI, comorbidity burden

Revision indications were balanced: aseptic loosening in 26 Type A (47.3%) versus 27 Type B (49.1%), periprosthetic fracture in 17 (30.9%) versus 15 (27.3%), post-infective reimplantation in 8 (14.5%) versus 9 (16.4%), and mechanical failure in four patients per group (7.3%). Operative time (156 ± 29 versus 160 ± 34 min; *p* = 0.41) and estimated blood loss (690 ± 220 versus 670 ± 205 mL; *p* = 0.57) were comparable.

Time to full weight bearing averaged 6.6 ± 2.0 weeks for Type A and 7.0 ± 2.3 weeks for Type B (*p* = 0.18). Patients who underwent ETO required slightly longer to achieve full weight bearing (mean 7.4 ± 2.3 weeks versus 6.5 ± 2.1 weeks for non-ETO), but the difference was not statistically significant (0.9-week mean difference; Cohen’s *d* = 0.42, 95% CI −0.00 to 0.84; *p* = 0.12). All osteotomies united uneventfully (mean 4.1 ± 0.7 months).

Radiographic assessment demonstrated high fixation rates. Osseointegration occurred in 52 Type A (94.5%) and 51 Type B (92.7%) stems. Mean distal stem subsidence was 1.3 ± 0.7 mm versus 1.5 ± 0.9 mm (*p* = 0.24), with early subsidence of 2–3 mm in 3 (5.5%) versus 4 (7.3%) patients. Minor, nonprogressive radiolucent lines (< 2 mm) were observed in 6 (10.9%) versus 5 (9.1%) cases. Limb-length restoration within 10 mm of the contralateral side was achieved in 49 (89%) Type A and 50 (90%) Type B cases, and femoral offset was restored in 47 (85%) versus 48 (87%).

Functional outcomes improved significantly in both groups. HHS increased from 44.7 ± 10.1 to 88.1 ± 7.0 (Type A) and 45.1 ± 9.6 to 87.3 ± 7.8 (Type B), OHS from 22.6 ± 6.5 to 40.9 ± 5.0 versus 22.0 ± 5.8 to 40.1 ± 4.9, and EQ-VAS from 55.2 ± 9.0 to 82.7 ± 7.5 versus 54.1 ± 8.6 to 81.3 ± 7.1 (all *p* < 0.001). Good-to-excellent outcomes (HHS > 80) were achieved in 46 (83.6%) Type A and 45 (81.8%) Type B patients. Patients who underwent ETO initially scored lower but reached similar final outcomes (Table 2).

**Table 2:** Operative, radiographic, and functional outcomes of the two groups

Outcome	Group A (*n* = 55)	Group B (*n* = 55)	Cohen’s *d*	95% CI	*p*-Value
Operative time (min), mean ± SD	156 ± 29	160 ± 34	−**0.13**	−0.50 to 0.25	0.41
Blood loss (mL), mean ± SD	690 ± 220	670 ± 205	** +0.09**	−0.28 to 0.47	0.57
Time to full weight bearing (weeks), mean ± SD	6.6 ± 2.0	7.0 ± 2.3	−**0.19**	−0.56 to 0.19	0.18
Osteotomy union (months), mean ± SD	4.1 ± 0.7	4.1 ± 0.7	**0.00**	−0.37 to 0.37	0.92
Osseointegration, *n* (%)	52 (94.5)	51 (92.7)			0.65
Stem subsidence (mm), mean ± SD	1.3 ± 0.7	1.5 ± 0.9	−0.25	−0.62 to 0.13	0.24
Early subsidence 2–3 mm, *n* (%)	3 (5.5)	4 (7.3)	—	—	0.69
Radiolucent lines < 2 mm, *n* (%)	6 (10.9)	5 (9.1)	—	—	0.75
Limb length restoration within 10 mm, *n* (%)	49 (89)	50 (90)	—	—	0.82
Femoral offset restored, *n* (%)	47 (85)	48 (87)	—	—	0.77
Harris Hip Score, mean ± SD	88.1 ± 7.0	87.3 ± 7.8	+0.11	−0.27 to 0.48	0.57
Oxford Hip Score, mean ± SD	40.9 ± 5.0	40.1 ± 4.9	+0.16	−0.21 to 0.54	0.46
EQ-VAS, mean ± SD	82.7 ± 7.5	81.3 ± 7.1	+0.19	−0.18 to 0.57	0.31
HHS > 80 (good–excellent), *n* (%)	46 (83.6)	45 (81.8)	—	—	0.78
Superficial infection, *n* (%)	3 (5.5)	2 (3.6)	—	—	0.65
Dislocation, *n* (%)	1 (1.8)	2 (3.6)	—	—	0.56
Late periprosthetic fracture, *n* (%)	1	1	—	—	1.00
5-year stem survivorship, % (95% CI)	98.2 (95.1–100)	97.6 (94.2–100)	—	—	0.71

Correlation analysis revealed a moderate negative association between time to full weight bearing and final HHS (*r* = −0.46, *p* < 0.01). Paprosky defect severity correlated with operative duration (*r* = 0.53, *p* < 0.001) and modestly with early subsidence. Multivariable regression identified earlier full weight bearing (*β* = −0.31, *p* = 0.01) and lower Paprosky grade (*β* = −0.27, *p* = 0.02) as independent predictors of final HHS, whereas stem type, ETO, and operative time were not predictive. The model explained 39% of HHS variability (adjusted *R*^2^ = 0.39). Logistic regression for HHS < 80 is detailed in Table 2.

Complications included superficial infection in 3 (5.5%) Type A and 2 (3.6%) Type B patients, all managed by debridement and antibiotics; dislocation in 1 (1.8%) versus 2 (3.6%), all managed by closed reduction with no redislocation in any patient. Late traumatic periprosthetic Vancouver C fracture occurred in 1 patient per group, both managed by fixation and stem retention. No deep infections, aseptic loosening, modular junction failures, or re-revisions occurred; however, given the relatively small sample size, these findings should be interpreted cautiously.

Five-year Kaplan–Meier stem survivorship was 98.2% (95% CI 95.1–100) for Type A and 97.6% (95% CI 94.2–100) for Type B. Since formal equivalence testing was not performed, no conclusion regarding statistical equivalence between the two stem types should be inferred.

## Discussion

Revision total hip arthroplasty (rTHA) remains one of the most technically demanding procedures in orthopedics. Surgeons are challenged not only by compromised femoral bone stock but also by the need to restore complex hip biomechanics. Over the past two decades, modular cementless long stems, such as the LIMA^™^ [22–30] and Revitan^™^ [1–20] systems, have transformed femoral reconstruction strategies, offering both modularity and secure diaphyseal fixation tailored to patient-specific anatomy and bone deficiency patterns. Unlike cemented systems, which rely on a uniform cement mantle and are vulnerable to metaphyseal bone loss, cementless modular tapered fluted stems exploit biological osseointegration to achieve a stable, adaptive bone–implant interface [13, 14, 22].

While both stems share the core principles of modularity, diaphyseal engagement, and distal fixation, subtle but meaningful differences exist. The LIMA^™^ stem features a tapered conical design with fluted geometry that promotes rotational stability and gradual stress transfer to the diaphysis. The modular junction allows independent adjustment of version, offset, and leg length, with optional distal locking in select cases [22–29]. In contrast, the Revitan^™^ stem employs a fluted, straight, or curved modular titanium design, with modular junctions designed for extreme length and version correction [1–20]. While Revitan^™^ has shown excellent survivorship in many series [1–7, 15], Dumoulin et al. reported a concerning rate of implant fractures with PFMR^®^ stems, highlighting the importance of balancing distal rigidity with bone preservation [1]. Although differences exist in taper, modular junction, and surface characteristics, our results indicate that these variations do not significantly affect mid-term clinical outcomes, including subsidence, osseointegration, or functional recovery.

The key advantages of modular tapered fluted stems include intraoperative adaptability, reliable distal fixation in poor metaphyseal bone, and the potential for early weight-bearing [19–22]. Disadvantages include technical complexity, risk of intraoperative femoral fracture, potential modular junction fretting/corrosion, and subsidence in severely osteoporotic bone [1, 15]. Understanding these distinctions is critical for surgical planning, implant selection, and patient counseling.

In our cohort, the LIMA^™^ modular long stem demonstrated durable fixation and significant functional recovery. Radiographs confirmed minimal subsidence, and no stem required revision for aseptic loosening, reflecting the synergistic effect of mechanical stability and biological integration. Functionally, all patients achieved meaningful improvements in Harris Hip Score, emphasizing that the combination of secure distal fixation and early mobilization optimizes both structural and biological recovery. These results are interpreted strictly on the basis of observed data and avoid subjective or philosophical descriptors of implant success.

When comparing our findings with the available literature on modular fluted tapered stems, consistent patterns emerge. Lorenzo Perticarini et al. reported reliable mid-term radiographic stability and significant functional improvement at approximately 6 years [23], while K. Zheng et al. demonstrated favorable outcomes even in cases of severe femoral bone loss, supporting durable fixation in complex revisions [24].

Long-term evidence further supports this concept, with Carl W. Park et al. showing excellent survivorship at a mean follow-up of 16 years [27]. Similarly, a narrative review by Fabio Randelli et al. highlighted the consistent reliability and low complication rates of these implants [29]. Moreover, Thibault Lucena et al. reported a low incidence of implant breakage, reinforcing their mechanical durability [30]. Across these studies, complications were consistently low: minor subsidence (< 5 mm), dislocations (3–5%), and occasional modular junction concerns (≤ 2%), mirroring our experience. Taken together, these studies collectively reinforce the reliability of modular long stems in diverse clinical scenarios, including massive bone loss, complex deformity, and extended trochanteric osteotomies [22–30]. Overall, these studies support the use of modular fluted tapered stems as a reliable option for achieving stable fixation, functional restoration, and acceptable complication rates in femoral revision arthroplasty.

Comparative insights with Revitan^™^ and other modular systems highlight both similarities and unique considerations. Both allow modular correction of version, offset, and leg length and emphasize diaphyseal fixation. Studies of Revitan^™^ stems report 5–10 year survivorship ranging from 91% to 98%, with functional gains comparable to our cohort [1–7, 15]. Key differences include junction design and distal fixation options, with Revitan^™^ sometimes demonstrating higher risk of distal stem fracture or subsidence in severe osteoporosis [1]. Importantly, our study demonstrates that, when applied under standardized surgical and rehabilitation protocols, both modular tapered fluted stems achieve outcomes that are statistically comparable in terms of radiographic stability, functional recovery, complication rates, and survivorship. Our results indicate comparable mechanical resilience of the LIMA^™^ stem to the Revitan^™^ stem without implying superiority beyond the data presented.

Cemented long-stem revisions remain a valid option in select cases, but limitations include risk of mantle fracture, early loosening, and compromised load distribution in bone-deficient femurs [11, 12]. Cementless modular tapered fluted stems, by contrast, exploit dynamic bone–implant interactions to achieve osseointegration, supporting both mechanical stability and long-term biological adaptation [13, 14].

To the best of our knowledge, this is the first study to directly evaluate and compare outcomes of the LIMA^™^ stem in a diverse cohort of rTHA patients, with detailed comparison to historical Revitan^™^ series and existing LIMA^™^ literature. While previous studies have individually reported survivorship, complications, and functional outcomes for either LIMA^™^ [22–30] or Revitan^™^ [1–20] stems, no prior study has systematically contextualized LIMA^™^ outcomes against alternative modular tapered fluted stems while integrating clinical, radiographic, and functional data.

This approach allows a comprehensive understanding of implant performance, mechanical resilience, and biologically driven osseointegration in complex femoral revisions, highlighting strengths and limitations of each system in real-world scenarios. The direct comparison confirms that subtle design differences do not significantly impact clinical outcomes, providing surgeons with evidence-based confidence in implant selection and reinforcing the clinical relevance of our findings for surgical planning and patient counseling.

Limitations include single-center experience, modest sample size, absence of direct randomized comparison with Revitan™ or other modular tapered fluted stems, and absence of a priori power calculation. Additional limitations are mid-term follow-up, limited ability to detect rare complications, and multiple comparisons without statistical correction, which may affect interpretation of results. While adjunctive procedures (e.g., extended trochanteric osteotomy) may influence early outcomes, our findings are consistent with international literature, confirming reproducibility with cementless modular tapered fluted stems.

## Conclusions

This study provides evidence that femoral revision arthroplasty can achieve successful outcomes when mechanical stability, modular adaptability, and biological osseointegration are optimized. To our knowledge, this is among the first studies to systematically contextualize outcomes of the LIMA^™^ stem within the landscape of alternative modular tapered fluted stems, providing direct comparative insights. Our results demonstrate that both modular tapered fluted stem systems achieve comparable mid-term survivorship, functional recovery, and low complication rates under standardized surgical and rehabilitation protocols.

These findings indicate that both implant designs provide reliable clinical and radiographic outcomes in complex femoral revision scenarios. However, the present study does not establish superiority of one system over the other and was not designed as a formal equivalence trial. Within these limitations, the results support the use of modular tapered fluted stems as versatile options for rTHA and provide clinically relevant data to assist surgeons in implant selection and surgical planning.

## Data Availability

The datasets used and/or analyzed during the current study available from the corresponding author on reasonable request.
